# Neurodiversity, Giftedness, and Aesthetic Perceptual Judgment of Music in Children with Autism

**DOI:** 10.3389/fpsyg.2017.01595

**Published:** 2017-09-22

**Authors:** Nobuo Masataka

**Affiliations:** Primate Research Institute, Kyoto University Inuyama, Japan

**Keywords:** autism spectrum disorder, music, aesthetic judgment, consonance, neurodiversity, Spielmann (wandering minstrel)

## Abstract

The author investigated the capability of aesthetic perceptual judgment of music in male children diagnosed with autism spectrum disorder (ASD) when compared to age-matched typically developing (TD) male children. Nineteen boys between 4 and 7 years of age with ASD were compared to 28 TD boys while listening to musical stimuli of different aesthetic levels. The results from two musical experiments using the above participants, are described here. In the first study, responses to a Mozart minuet and a dissonant altered version of the same Mozart minuet were compared. In this first study, the results indicated that both ASD and TD males preferred listening to the original consonant version of the minuet over the altered dissonant version. With the same participants, the second experiment included musical stimuli from four renowned composers: Mozart and Bach’s musical works, both considered consonant in their harmonic structure, were compared with music from Schoenberg and Albinoni, two composers who wrote musical works considered exceedingly harmonically dissonant. In the second study, when the stimuli included consonant or dissonant musical stimuli from different composers, the children with ASD showed greater preference for the aesthetic quality of the highly dissonant music compared to the TD children. While children in both of the groups listened to the consonant stimuli of Mozart and Bach music for the same amount of time, the children with ASD listened to the dissonant music of Schoenberg and Albinoni longer than the TD children. As preferring dissonant music is more aesthetically demanding perceptually, these results suggest that ASD male children demonstrate an enhanced capability of aesthetic judgment of music. Subsidiary data collected after the completion of the experiment revealed that absolute pitch ability was prevalent only in the children with ASD, some of whom also possessed extraordinary musical memory. The implications of these results are discussed with reference to the broader notion of neurodiversity, a term coined to capture potentially gifted qualities in individuals diagnosed with ASD.

## Introduction

Neurodiversity refers to the notion that seemingly ‘impaired’ cognitive as well as emotional properties characteristic of developmental disorders such as autism spectrum disorders (ASD) a neurodevelopmental disorder with unusual sensory processing, are not necessarily deficits, but fall into normal behavioral variations exhibited by humans. Stated more formally, this notion was recently described as “a concept where neurological differences are to be recognized and respected as any other human variation” (Armstrong, 2012). The term was first coined in the late 1990s by New York journalist Harvey Blume and Australian autism activist Judy Singer, and has become an important component of the civil rights movement for those with neurologically based disabilities. While this is indeed a paradigm shift from “deficit-oriented view of ASD” to “strength-oriented view of ASD” (Silberman, 2015), experimental and empirical scientific evidence confirming this conceptual notion and paradigm has been meager.

From the archeological perspective, however, a recent review mentioned the enhanced perceptual abilities of the individuals with this disorder and argues the possibility that these might contribute the survival of the individuals in a Paleolithic context (Spikins et al., 2016). From a cognitive science perspective, on the other hand, here the author presents experimental evidence indicating the gifted cognitive capability of such individuals and suggests the historical presence of a group of professional individuals who had relied upon the capability to survive, being incorporated into human societies. That concerns with the capability of aesthetic judgment of music.

As noted by Temple Grandin (Grandin, 1996; Grandin and Cook, 2004), a strong appreciation for music is commonly observed in children with ASD. Kanner (1943) has already reported extraordinary musical memory in 6 of 11 individuals who were diagnosed as this disorder. The first systematic attempt to identify superior performance on a musical task found that reproduction of atonal melodies was superior in children with ASD, as compared IQ-matched, typically developing (TD) children (Applebaum et al., 1979). Since that study, increased sensitivity to musical pitch and timbre (Heaton et al., 1998), including absolute pitch (AP), has been documented frequently in children with ASD (see Heaton, 2009 for review).

So far, such atypical musical skills have been interpreted in terms of enhanced perceptual processing of lower-order structure of music that is characteristic of this disorder on the assumption that the superiority should not extend to the cognitive sense of music in children with ASD (Mottoron et al., 2000, 2006). However, recently, neurological evidence against the assumption has been presented (Gebauer et al., 2014). In that study, neural correlates of emotional response to music were compared between adults with ASD and neurotypical controls, using functional magnetic resonance imaging. The results revealed that both groups engaged similar neural networks during processing of emotional music. However, in the ASD group, increased activity in response to happy compared to sad music was observed in dorsolateral prefrontal regions as well as in the rolandic operculum/insula, indicating enhanced cognitive processing and physiological arousal in response to emotional musical stimuli in this group. These findings suggest the possibility that an aesthetic emotional response *per se* somehow occurs atypically upon listening to music in individuals with ASD, among whom there are known to be some remarkably musically skillful individuals. This is the issue pursued in the current study.

As a first step, here, the author has compared the aesthetic judgments of consonant/dissonant melody between children with ASD and TD children because this is the issue that has been most intensively as to spontaneous preference for some specific patterns of music. In the Western world as well as in Japan, even newborns are known to show preference for a Mozart’s minuet, most of which consists of consonant intervals, over a modified version of it that mostly consists of dissonant intervals (Trainor and Heinmiller, 1998; Masataka, 2006). Thus, the identical testing was undertaken first here with children with ASD and TD children.

While consonant intervals are most often used among popular music around the world, it is also true that some pieces of Western classical music are highly dissonant (Boulez, 1971; Masataka, 2003). Nevertheless, they are appreciated as a source of pleasure. Darwin (1871) already mentioned this fact and was cautious about the connection between musical consonance and hedonicity. For instance, sad and dissonant music like Schoenberg’s piano pieces and Adagio by Alibinoni certainly evoke some aesthetic emotions in individuals exposed to them (Boulez, 1971; Thompson et al., 2001; Masataka and Perlovsky, 2013). However, such a pleasurable experience may require a sensitivity that needs to be cultivated as it develops in a particular music culture, unlike preferences for a Mozart’s minuet over its modified version with more dissonant intervals. Given this possibility, the degree of the preference for these music pieces in a child, if it is measurable, would reveal the degree to which the musical intelligence defined by the theory of multiple intelligences (Gardner, 2011) has developed in the child. Along such reasoning, in the second experiment of the current study, preferences for such works as well as for typical consonant music such as Mozart’s and Bach’s pieces were investigated in the two groups of participants.

In order to confirm the major findings of previous studies about unusual musical sensitivity of children with ASD, subsidiary data were collected after the completion of the above experiments from all the participants about AP, the ability to identify the frequency or musical name of a specific tone, or to identify a tone without comparing it with any objective reference tone (Masataka, 2011). The parents of the participants were also interviewed to ask about instances of extraordinary musical memory in their children, which has been reported to occur often in association with ASD (Kanner, 1943).

## Materials and Methods

This investigation was conducted according to the principles expressed in the Declaration of Helsinki. All experimental protocols were consistent with the Guide for Experimentation with Humans, and were approved by the Institutional Ethics Committee, of the Primate Research Institute, Kyoto University (#2011-150). The authors obtained written informed consent from parents of all participants involved in the study.

### Participants

A group of 19 male children with ASD aged 4–7 years (*M* = 5.8; *SD* = 1.2) and 28 TD male children aged 4–7 years (*M* = 5.6; *SD* = 1.4) were studied in the current study. All participants were musically untrained. There was no significant difference between the mean age of the two participant groups [*t*(45) = 1.10, *p* = 0.28]. All participants were Japanese, right-handed, naïve as to the purpose of this study, and auditorily normal.

Nineteen children with ASD were recruited for the current study. Based on direct clinical observation of each child by an independent child psychiatrist, a diagnosis of autism was made according to ICD-10 (World Health Organization, 1994) as well as DSM-IV (American Psychiatric Association, 1994). On the basis of such criteria, each participant in the group of children with ASD was diagnosed as either F84.0, F84.9, or F84.8. Moreover, such diagnoses were also confirmed by the Autism Diagnostic Interview-Revised (ADI-R), an extensive, semi-structured parental interview (Lord et al., 1994) that was conducted by an independent psychiatrist. The ADI-R provides information about the presence of verbal language skills, defined as daily, functional and comprehensive use of spontaneous phrases of at least three words and occasionally a verb. All of the participant ASD children were found to express verbal language. All of the TD children were recruited via the board of education in a small city in Japan. All participants attended normal classes characteristic of their chronological age level. None of the participants included in the groups of TD children met any diagnostic criterion for autism or any other pervasive developmental disorder.

### Procedure

Throughout the current study, two experimental trials were executed to investigate music preference of each participant (referred to as “Experiment 1” and “Experiment 2” below), using the same experimental protocol but different materials as stimuli. None of the participants had been exposed to any of the materials prior to the current study.

The materials used in Experiment 1 were the original, harmonic version of a Mozart’s simple minuet (C major K.#1f) and its modified, inharmonic version. They were essentially the same as used previously (Trainor and Heinmiller, 1998; Masataka, 2006; Masataka and Perlovsky, 2012, 2013). Both the original and the modified versions were digitally generated and created by piano timbre. They were made up of 60 intervals. In the original, harmonic version, only three of them were dissonant, and all three were tritons (6-semitone intervals). In the modified, inharmonic version, all Gs were changed to F#s and all Ds to C#s. This had the effect of creating 21 additional dissonant intervals, including a total of 12 of the two most-dissonant intervals, i.e., seven tritons and five minor ninths (13 semitones). In the present stimuli, the upper voice and the lower voice were separated by more than an octave in each interval. The tempo was identical across the two versions.

The materials used in Experiment 2 were four musical pieces, namely, Mozart’s piano sonata K.448, Bach’s toccata in G major BWV 916, Schoenberg’s Klavierstuek op.33a, and Adagio in G minor for organ and strings by Albinoni. While listening to these musical pieces of Mozart and Bach has been reported to enhance spatial tasks, referred to as Mozart effect (Cooper, 2001), the musical pieces of Schoenberg and Albinoni used here are well known as representative of the most popular pieces of highly dissonant western classical music.

As the protocol, the current study adapted the child contingent head-turn preference procedure to allow musical preferences to be measured by the participant pressing a key in order to hear a given extract of music (from a choice of either of two keys/musical pieces in Experiment 1 and from a choice of four possible keys/musical pieces in Experiment 2). The data thus show discrimination among a number of musical styles, and preferences can also be measured. Participants were tested individually, sitting in a quiet room under daylight conditions in front of a table. There was a toy keyboard equipped with eight keys on the table. When a participant entered the room and was seated, an experimenter who was sitting apart from him instructed him that some of the keys would start flashing and that he could hear something by pressing either of them. The keyboard was connected to a portable computer with specially written software, enabling the presentation of each of the prepared stimuli to be linked to each of the flashing keys and to be heard only when the key is pressed. Ten seconds after the instruction, two of the keys started flashing and preliminary trials began. When the participant pressed either of them, a popular Japanese playsong was played. It continued to be played as long as he kept pressing. However, nothing was played when he pressed others keys or more than one key simultaneously. By conducting such trials for a maximum of a 5-min period, each participant learned to perform what was necessary to listen to prepared music stimuli. After the experimenter had confirmed the learning, Experiment 1 was started after a 1-min break. It was 5-min long, and was followed by Experiment 2 of 10-min length after another 1-min break. Thereafter, the participant was instructed to leave the testing chamber. During each break, none of the keys flashed.

In Experiment 1, only two keys of the eight were to flash lights, while four of them were to flash in Experiment 2. The relation of each of the keys and the type of stimulus used was fixed in each experimental trial involving a given participant, randomly determined across the participants, and counterbalanced. The keys were kept flashing during Experiments 1 and 2. During that period, he was allowed to press any key. As long as he kept pressing either of the flashing keys, he was exposed to the music assigned to that key (sound pressure level: 60 dB). However, the music was never played for duration longer than 1 min. If he kept pressing the key longer than 1 min, the computer ceased to present the stimulus. Key presses were recorded in msec, and preference for each music stimulus was calculated as a proportion of the total time allocated to the stimulus.

In testing of AP ability (referred to as Experiment 3), the participants heard 36 pure sine wave tones, presented in pseudorandomized order, which ranged from A3 (tuning: A4 = 440 Hz) to A5, with each tone being presented once. Each tone of the AP test had a duration of 1 s, with a 6-s interstimulus interval. The participants had to answer the tonal label after hearing the accordant tone. Prior to the testing, each participant had been confirmed to have acquired sufficient verbal knowledge about the label to answer. The whole test unit and its components were created with Adobe Audition 1.5. The accuracy was evaluated by counting correct responses. The participants were not asked to identify the adjacent octaves of the presented tones because for AP, identifying the correct chroma is a most notable prerequisite.

In interviews with the parents of the participants, episodes of two children with ASD documented by Kanner (1943) were mentioned; Charles N. was able to distinguish among 18 symphonies before he was 2 years old, and when his mother played his favorite records, he could answer the name of the symphony heard correctly. Another boy, John F., was similarly talented at recognizing melodies. If his father whistled a tune, he often identified it, for instance, as Mendelssohn’s violin concerto. In addition, he was able to recite many prayers and nursery rhymes from memory. After such episodes were mentioned to them, the parents in the present study were asked whether they had experienced similar instances in their children.

## Results

**FIGURE 1:**
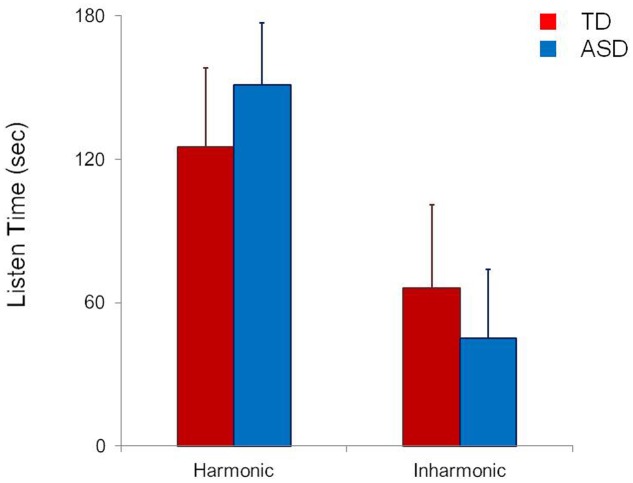
Mean preference (error bars: SDs) for an original simple harmonic minuet of Mozart (Harmonic) and its modified inharmonic version containing many dissonant intervals (Inharmonic) in children with autism spectrum disorder (ASD) and in typically developing (TD) children.

The overall results of Experiment 1 are summarized in **Figure 1**, which shows the overall mean duration of listening to the original, harmonic version of the Mozart’s minuet and to its modified, inharmonic version with many dissonant intervals in the group of TD children and that of ASD children. When the collected data were analyzed using a 2 (ASD/TD, PARTICIPANT) × 2 (original version versus modified version, STIMULUS) analysis of variance (ANOVA), one of the two main effects (STIMULUS) was statistically significant [*F*(1,45) = 49.53, *p* = 0.0000, $\eta_{p}^{2}$ = 0.299]. The other main effect (PARTICIPANT) was not significant [*F*(1,45) = 0.84, *p* = 0.46, $\eta_{p}^{2}$ = 0.033]. However, the interaction between PARTICIPANT and STIMULUS was also significant [*F*(1,45) = 4.25, *p* = 0.028, $\eta_{p}^{2}$ = 0.35].

Subsequent analyses of simple main effects (Bonferroni correction) revealed that the mean listening time was shorter in response to the inharmonic version than to the harmonic version in both the children with ASD and TD children (*p* < 0.001). Moreover, the children with ASD listened to the harmonic version longer than the TD children (*p* = 0.03), who listened to the inharmonic version longer than the children with ASD (*p* = 0.04).

**FIGURE 2:**
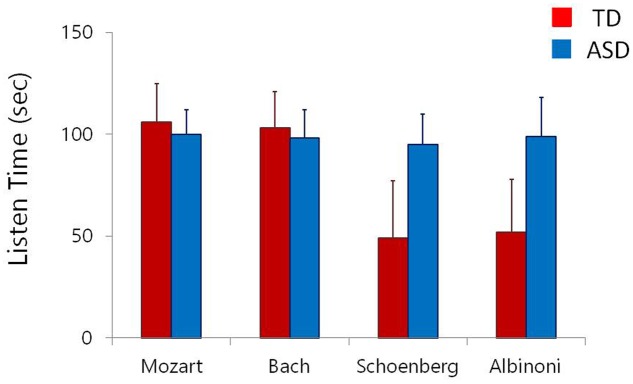
Mean preference (error bars: SDs) for musical pieces of Mozart, Bach, Schoenberg and Albinoni in children with autism spectrum disorder (ASD) and in typically developing (TD) children.

The overall results of Experiment 2 are summarized in **Figure 2**, which shows the overall mean duration of listening to the pieces of music from the four classic works in the group of TD children and that of ASD children. When the data were analyzed using another ANOVA, both of the two main effects were statistically significant [*F*(1,45) = 48.33, *p* = 0.0000, $\eta_{p}^{2}$ = 0.287 for STIMULUS and *F*(1,45) = 9.12, *p* = 0.009, $\eta_{p}^{2}$ = 0.133 for PARTICIPANT]. The interaction between PARTICIPANT and STIMULUS was also significant [*F*(3,135) = 4.25, *p* = 0.012, $\eta_{p}^{2}$ = 0.30].

Pair-wise comparisons of the mean listening time across the four different music stimuli in the TD children and the children with ASD revealed that the TD children listened longer to both Mozart’s piece and Bach’s piece than to either Schoenberg’s piece or Albinoni’s piece (*p*s < 0.01). Besides these, no such differences were found with regard to any pair among the four stimuli (*p*s > 0.10). The mean listening time of the children with ASD to any of the four stimuli did not significantly differ from that to any of the remaining three stimuli (*p*s > 0.10). Overall the children with ASD listened to Schoenberg’s piece and Albinoni’s piece longer than the TD children (*p*s < 0.01) whereas the mean listening time did not differ significantly between the two participant groups with regard to Mozart’s piece or Bach’s piece (*p*s > 0.10).

**FIGURE 3:**
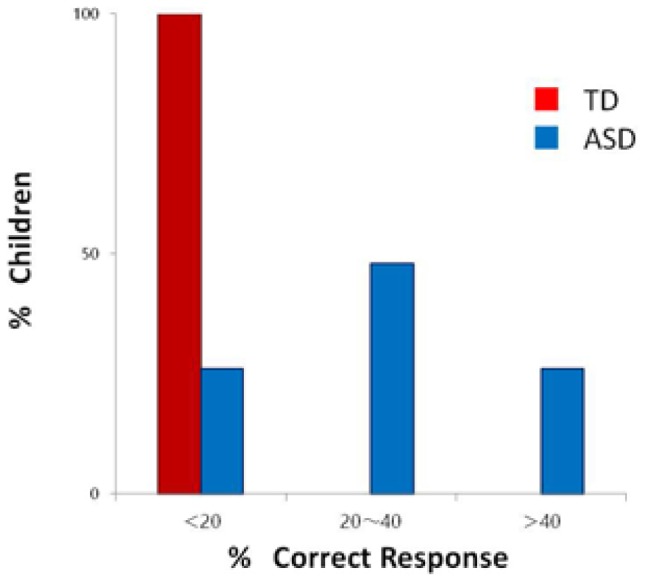
Accuracy scores in absolute pitch testing in children with autism spectrum disorder (ASD) and in typically developing (TD) children.

The overall results of the subsequent AP testing (Experiment 3) are presented in **Figure 3**. While the accuracy scores of all of the TD children remained within the level of chance, those of 15 of the 19 children with ASD were found to exceed the level of chance. During the interviews with the parents of the participants, some instance of extraordinary musical memory in their children was reported by six parents of the children with ASD, but there was no such report by any parent of the TD children.

## Discussion

Regarding TD children, the results of Experiment 1 of the current study are consistent with those reported previously (Trainor and Heinmiller, 1998; Masataka, 2006) and these participants exhibited a preference for the original, harmonic version of a Mozart’s minuet over its modified version containing many dissonant intervals. The results of this experiment also showed the fact that such a preference was confirmed in children with ASD. Moreover, the extent of the preference was more robust in children with ASD than in TD children, indicating that children with ASD are more sensitive than TD children are with regard to the aesthetic judgment of consonance and dissonance *per se* in music.

The results of Experiment 2 are seemingly to contradictory to those of Experiment 1. Namely, children with ASD listened to the music stimuli indiscriminately whether the music consisted of many dissonant intervals or not whereas TD children listened to the pieces of music consisting of many consonant intervals longer than to those consisting of dissonant intervals, to which the TD children actually avoided listening. Due to such avoidance, the total duration of listening to the stimuli was longer in children with ASD than in TD children. However, it should be noted that the pieces of music containing many dissonant intervals that were used as the stimuli in Experiment 2 were from famous composers of Western classical music. These pieces contained such dissonant intervals as a consequence of the composers’ attempt to aesthetically express the emotion of sadness, whereas in Experiment 1, Mozart’s original minuet was merely modified using a means to create its dissonant version.

This difference differentially influenced the responses to the stimuli in children with ASD, but not in TD children. This fact appears to deserve further analysis. Possibly, it may point to a question about the validity of the general assumption that the relative proportion between consonance and dissonance in music corresponds to a hedonic division between positive and negative that, apparently, in turn corresponds to the distinction between major and minor in music. The present results suggest that children with ASD possess enhanced capability of aesthetic judgment and appreciated the presented pieces of classical music even though they contained many dissonant intervals. This, in turn, indicates that it would be difficult to explain the more robust response to consonance/dissonance difference in children with ASD than in TD children found in Experiment 1 merely in terms of an enhanced perceptual processing of lower-order structure of music that is associated with ASD.

Among a number of acoustical dimensions constituting music, that of consonance/dissonance is the developmentally earliest that can be judged. During early childhood, neurotypical individuals are likely to listen to harmonic music preferably to music with many dissonant intervals even if those intervals in the latter music were a consequence of an artistic attempt to express the emotion of sadness aesthetically (Winner, 2006). Appreciation of sad music develops later. While this was indeed experimentally confirmed in Experiment 2 of the current study, the present results also raise the possibility that children with ASD who are aged-matched to TD children possess a more developed capability of aesthetic judgment of music even though musically untrained.

Along with the conceptualization of the theory of multiple intelligences (Gardner, 2011), the cognitive characteristics revealed in children with ASD can be explained as greater than typical strength of musical intelligence in themselves although the children experience difficulty with interpersonal communication (Lord et al., 1994; World Health Organization, 1994). While the pattern of operation of an individual person’s mind can be categorized according to the domain toward which that individual is more oriented, individuals with ASD, overall, do not rely upon their social relationships but rather are predisposed to process perceived non-social objects in more depth (Masataka, 2017). For instance, these individuals “exhibit enhanced discrimination between auditory stimuli, more accurate local target detection of auditory stimuli, and diminished global interference with auditory processing.” These abilities, referred to as “naturalist intelligence” by Gardner (2011) are highly adaptive for living in nature, and in particular, would seem to have conferred an evolutionary advantage upon individuals during prehistoric times, as well as upon individuals in later times who would prefer to live outside any community (Armstrong, 2017).

The enhanced capability of aesthetic judgment of music found here was associated with some atypical musical skills characteristic of ASD such as AP and extraordinary musical memory, as revealed by the subsidiary data collected after the completion of Experiments 1 and 2. These skills are known to have been possessed by an outstanding artist in the 16th century, Wolfgang Mozart, who could name the note on hearing a bell toll, or a clock or even a pocket watch strike, and could memorize a 10-min-long melody that he heard only once (Deutsch, 2006). It should also be noted that his family kept continually touring throughout Europe from the time he was 5 years until 21 years of age, which contributed to establishing the young composer’s reputation as a musical prodigy. Such a lifestyle was indeed in accord with the lifestyles of such individuals who were documented in historical records in Europe as professional musical performers (Hartung, 2003).

The documentation of such individuals in southern Europe, the region between the Alps and the Pyrenees, appeared as early as the 10th century, with them being referred to as “Spielmann (plural: Spielleute)” in German and as “wandering minstrels” in English. Spielleute had no settled abode, and instead used to roam about from place to place over a broad range of regions across a number of communities. In the 13th century, such individuals came to be distributed over all regions of Europe. While all other individuals who were integrated into feudal society were occupied with predetermined, inherited work, Spielleute were the first who had chosen this occupation of their own will. Born in urban regions in the society, most of them felt difficulty in staying there and decided to live as outcasts. As a result, they were treated as being dishonorable by society members. They were talented in a variety of rudimentary forms of contemporary art performance, performing arts including music and dancing without experiencing any form of professional training. In particular, they were well known to perform mimicking of a variety of sounds heard in the landscape (such as bird songs) while playing stringed and/or woodwind instruments simultaneously (Hartung, 1982). When one of their performances was successful, the melody was memorized no matter how long it was without recording it using musical notation (actually, they were unable to read music) and it became part of their repertoire of routine performance. Such a seemingly mysterious endowment seems also to have been possessed by some famous modern artists who contributed to the subsequent development of classical music, such as Mozart, Bach and Beethoven (Deutsch, 2006), and also by the children with ASD in the current study.

Spielleute were allowed to travel around as they liked by the churches and the lords of communities, their daily income totally depending upon their performances. They were apparently regarded as exceptional individuals (outsiders). The official position of the Catholic church was that they lived in a state of sin (Stuart, 1999). Nevertheless they used to play an important role, i.e., the role of ‘negotiator’ once conflict happened between regional communities within feudal society because they were familiar with members of both of the disputing communities. That indicates that despite being socially marginalized, such individuals were socially functional. While “Spielleute” or “wandering minstrels” were neurdivergent individuals who came to be no longer recorded after the 18th century as Europe became industrialized, the fact should be noted that similar outcast groups of individuals have been commonly documented among a variety of cultures and that all of them were talented in musical performance without exception (Hartung, 2003). That again strongly suggests the possibility that they possessed an enhanced aesthetic sense of music such as the giftedness that was revealed here in the performance of children with ASD.

As clinical implications of the findings, one can also mention the possibility that this early onset and/or potentially predispositional demonstration of heightened aesthetic judgment of music might eventually lead to new job-related opportunities for individuals with ASD in uniquely useful and professionally applicable settings in new fields. No doubt, this is an issue that should be clinically addressed in the next step of research.

## Author Contributions

NM designed the study, collected and analyzed the data and drafted the manuscript.

## Conflict of Interest Statement

The author declares that the research was conducted in the absence of any commercial or financial relationships that could be construed as a potential conflict of interest.

## References

[B1] American Psychiatric Association (1994). *Diagnostic and Statistical Manual ofMental Disorders.* Washington DC: American Psychiatric Association, 1–609.

[B2] Applebaum E. Egel A. L. Koegel R. L. Imboff B. (1979). Measuring musical abilities of autistic children. *J. Autism Dev. Disord.* 9 279–285. 10.1007/BF01531742 489514

[B3] Armstrong T. (2012). *Neurodiversity in the Classroom: Strength-based Strategies to Help Students with Special Needs Succeed in School and Life.* Alexandria, VA: ASCD, 1–183.

[B4] Armstrong T. (2017). The healing balm of nature: understanding and supporting the naturalist intelligence in individuals diagnosed with ASD. *Phys. Life Rev.* 20 109–111. 10.1016/j.plrev.2017.01.012 28117238

[B5] Boulez P. (1971). *Boulez on Music Today.* London: Faber and Faber, 1–156.

[B6] Cooper J. S. (2001). The mozart effect. *J. R. Soc. Med.* 94 170–172. 11317617 10.1177/014107680109400404 PMC1281386

[B7] Darwin C. R. (1871). *The Descent of Man, and Selection in Relation to Sex.* London: John Murray, 1–265.

[B8] Deutsch D. (2006). The enigma of absolute pitch. *Acoust. Today* 2 11–19. 10.1121/1.2961141

[B9] Gardner H. (2011). *Frame of Mind: The Theory of Multiple Intelligences.* New York, NY: Basic Books, 1–387.

[B10] Gebauer L. Skewes J. Westphael G. Heaton P. Vuust P. (2014). Intact brain processing of musical emotions in autism spectrum disorder, but more cognitive load and arousal in happy vs sad music. *Front. Neursci.* 8:192 10.3389/fnins.2014.00192 PMC4098021 25076869

[B11] Grandin T. (1996). *Thinking in Pictures.* New York, NY: Vintage, 1–177.

[B12] Grandin T. Cook K. (2004). *Developing Talents: Careers for Individuals with Asperger Syndrome and High-functioning Autism.* Lenexa, KS: Autism Asperger Publishing, 1–185.

[B13] Hartung W. (1982). *Die Spielleute: Eine Randgruppe in der Gesellschaft des Mittelalters.* Wiesbaden: Franz Steiner Verlag, 1–108.

[B14] Hartung W. (2003). *Die Spielleute im Mittelalter: Gaukler, Dichter, Musikanten.* Dusseldorf: Artemis & Winkler, 1–405.

[B15] Heaton P. (2009). Assessing musical skills in autistic children who are not savants. *Philos. Trans. R. Soc. B* 364 1443–1447. 10.1098/rstb.2008.0327 PMC2677585 19528029

[B16] Heaton P. Hermelin B. Pring L. (1998). Autism and pitch processing: a precursor for savant musical ability? *Music Percept.* 15 291–305. 10.2307/40285769

[B17] Kanner L. (1943). Autistic disturbances in affective contact. *Nerv. Child* 2 217–250.

[B18] Lord C. Rutter M. Le Couteur A. (1994). Autism diagnostic interview-revised: a revised version of a diagnostic interview for caregivers of individuals with possible pervasive developmental disorders. *J. Autism Dev. Disord.* 24 659–685. 10.1007/BF02172145 7814313

[B19] Masataka N. (2003). *The Onset of Language.* Cambridge: Cambridge University Press, 1–281. 10.1017/CBO9780511489754

[B20] Masataka N. (2006). Preference for consonance over dissonance by hearing newborns of deaf parents and of hearing parents. *Dev. Sci.* 9 46–50. 10.1111/j.1467-7687.2005.00462.x 16445395

[B21] Masataka N. (2011). Enhancement of speech-relevant auditory acuity in absolute picth possessors. *Front. Psychol.* 2:101 10.3389/fpsyg.2011.00101 PMC3132675 21779258

[B22] Masataka N. (2017). Implications of the idea of neurodiversity for understanding the origins of developmental disorders. *Phys. Life Rev.* 20 85–108. 10.1016/j.plrev.2016.11.002 27876343

[B23] Masataka N. Perlovsky L. (2012). The efficacy of musical emotions evoked by Mozart’s music for the reconciliation of cognitive dissonance. *Sci. Rep.* 2 307 10.1038/srep00307 PMC3457076 23012648

[B24] Masataka N. Perlovsky L. (2013). Cogntive interefernce can be mitigated by consonant music and facilitated by disoonant music. *Sci. Rep.* 3:2028 10.1038/srep02028 PMC3685829 23778307

[B25] Mottoron L. Dawson M. Soulieres L. Hubert B. Burack J. (2006). Enhanced perceptual functioning in autism: An update, and eight principles of autistic perception. *J. Autism Dev. Disord.* 2 1–17. 10.1007/s10803-005-0040-7 16453071

[B26] Mottoron L. Peretz I. Menard E. (2000). Local and global processing of music in high-functioning persons with autism: beyond central coherence? *J. Child Psychol. Psychiatry* 41 1057–1065. 10.1111/1469-7610.00693 11099122

[B27] Silberman S. (2015). *Neurotribes: The Legacy of Autism and the Future of Neurodiversity.* New York, NY: Avery, 1–534.

[B28] Spikins P. Wright B. Hodgson D. (2016). Are there alternative adaptive strategies to human pro-sociality? The role of collaborative morality in the emergence of personality variation and autistic traits. *Time Mind* 9 289–313. 10.1080/1751696X.2016.1244949

[B29] Stuart K. (1999). *Defiled Trades Social Outcasts: Honor and Ritual Pollution in Early Modern Germany.* Cambridge: Cambridge University Press, 1–286.

[B30] Thompson W. F. Schellenberg E. G. Husain G. (2001). Arousal mood and the Mozart effect. *Psychol. Sci.* 12 248–251. 10.1111/1467-9280.00345 11437309

[B31] Trainor L. Heinmiller B. M. (1998). The development of evaluative response to music: Infants prefer to listen to consonance over dissonance. *Infant Behav. Dev.* 21 77–88. 10.1016/S0163-6383(98)90055-8

[B32] Winner E. (2006). “Development in the arts: drawing and music,” in *Handbook of Child Psychology* Vol. 2 ed. Damon R. (New York, NY: Wiley), 859–904.

[B33] World Health Organization (1994). *The Composite International Diagnostic Interview, Version 1.1.* Geneva: World Health Organization, 1–632.

